# Childhood pneumonia increases risk for chronic obstructive pulmonary disease: the COPDGene study

**DOI:** 10.1186/s12931-015-0273-8

**Published:** 2015-09-21

**Authors:** Lystra P. Hayden, Brian D. Hobbs, Robyn T. Cohen, Robert A. Wise, William Checkley, James D. Crapo, Craig P. Hersh, on behalf of the COPDGene Investigators

**Affiliations:** Division of Respiratory Diseases, Boston Children’s Hospital, 300 Longwood Ave., Boston, MA 02115 USA; Channing Division of Network Medicine, Brigham and Women’s Hospital, 181 Longwood Ave., Boston, MA 02115 USA; Division of Pulmonary and Critical Care Medicine, Brigham and Women’s Hospital, 75 Francis St., Boston, MA 02115 USA; Department of Pediatrics, Boston University School of Medicine, 72 E Concord St., Boston, MA 02118 USA; Division of Pulmonary and Critical Care, Johns Hopkins University School of Medicine, 1830 E. Monument St., Baltimore, MD 21205 USA; Department of Medicine, National Jewish Health, 1400 Jackson St., Denver, CO 80206 USA

## Abstract

**Background:**

Development of adult respiratory disease is influenced by events in childhood. The impact of childhood pneumonia on chronic obstructive pulmonary disease (COPD) is not well defined. We hypothesize that childhood pneumonia is a risk factor for reduced lung function and COPD in adult smokers.

**Methods:**

COPD cases and control smokers between 45–80 years old from the United States COPDGene Study were included. Childhood pneumonia was defined by self-report of pneumonia at <16 years. Subjects with lung disease other than COPD or asthma were excluded. Smokers with and without childhood pneumonia were compared on measures of respiratory disease, lung function, and quantitative analysis of chest CT scans.

**Results:**

Of 10,192 adult smokers, 854 (8.4 %) reported pneumonia in childhood. Childhood pneumonia was associated with COPD (OR 1.40; 95 % CI 1.17-1.66), chronic bronchitis, increased COPD exacerbations, and lower lung function: post-bronchodilator FEV_1_ (69.1 vs. 77.1 % predicted), FVC (82.7 vs. 87.4 % predicted), FEV_1_/FVC ratio (0.63 vs. 0.67; *p* < 0.001 for all comparisons). Childhood pneumonia was associated with increased airway wall thickness on CT, without significant difference in emphysema. Having both pneumonia and asthma in childhood further increased the risk of developing COPD (OR 1.85; 95 % CI 1.10-3.18).

**Conclusions:**

Children with pneumonia are at increased risk for future smoking-related lung disease including COPD and decreased lung function. This association is supported by airway changes on chest CT scans. Childhood pneumonia may be an important factor in the early origins of COPD, and the combination of pneumonia and asthma in childhood may pose the greatest risk.

**Clinical trials registration:**

ClinicalTrials.gov, NCT00608764 (Active since January 28, 2008).

**Electronic supplementary material:**

The online version of this article (doi:10.1186/s12931-015-0273-8) contains supplementary material, which is available to authorized users.

## Background

Pneumonia is a common pediatric diagnosis that poses a significant risk for future respiratory disease [1, 2]. Multiple investigations have found an association between pneumonia in childhood and decreased adult lung function, raising the question of whether childhood pneumonia is a risk factor for chronic obstructive pulmonary disease (COPD). Prior studies are limited by small sample sizes, short-term follow-up, absence of post-bronchodilator lung function, differing definitions of respiratory illness, sampling bias, and recall bias [3–10]. This study examines the effect of childhood pneumonia in a large population of older adults, including objective diagnosis of COPD with standardized post-bronchodilator spirometry and analysis of chest computed tomography (CT).

Smoking remains a major risk for children, with one in fifteen high school seniors reporting daily cigarette use [11]. Most smokers initiate the habit by age 18, putting them at risk for a wide range of comorbidities including COPD [12]. Recently, there has been interest in the early origins of COPD and the potential synergistic relationship between childhood respiratory infection, aberrant lung development, and increased susceptibility to smoking related injury [10, 13–15]. More data is needed to guide providers in anticipating the outcomes of childhood pneumonia and potential additional complications from smoking.

This study examines the association between pneumonia in childhood and future respiratory illness in smokers. We hypothesize that childhood pneumonia is a risk factor for reduced lung function and COPD in adult smokers. Some of the results have been published previously as an abstract [16].

## Materials and methods

### Subjects

We evaluated 10,192 current and former United States smokers with and without COPD from the COPDGene Study, a multicenter, observational study designed to identify genetic and environmental factors associated with COPD. COPDGene enrolled subjects from 2008–2011. It was approved by the Institutional Review Boards at each of the twenty-one clinical sites. All participants provided written informed consent [17]. Subjects were 45–80 years of age, non-Hispanic white or African American, and had at least a 10 pack-year smoking history. Exclusion criteria included history of lung disease other than COPD or asthma (e.g. extensive bronchiectasis, cystic fibrosis, pulmonary fibrosis, lung cancer). Study protocol, enrollment criteria, and data collection forms were previously described and are available at www.copdgene.org [17, 18].

### Data collection

Participants completed a modified American Thoracic Society Respiratory Epidemiology Questionnaire, Modified Medical Research Council (MMRC) dyspnea scale, and questionnaires related to demographics and medical history [18–20]. Quality of life was assessed using the St. George’s Respiratory Questionnaire (SGRQ) [21]. Subjects completed a standardized spirometry protocol (ndd EasyOne Spirometer, Zurich, Switzerland). Inspiratory and expiratory chest CT scans were obtained. Airway measurements, performed using VIDA software (VIDA Diagnostics; Iowa City, Iowa), assessed wall thickening in segmental airways, subsegmental airways, and the square root of the wall area of a hypothetical airway with 10mm internal perimeter (SRWA-Pi10) [22, 23]. SLICER software (www.slicer.org) was used to quantify emphysema by inspiratory scan low-attenuation areas < -950 Hounsfield units (HU) and gas trapping on expiratory scan at < -856 HU [24].

### Case identification

Childhood pneumonia was defined by subject self-report. The questionnaire asked: “Have you ever had pneumonia or bronchopneumonia?” and their age at the first episode. Subjects were classified as childhood pneumonia if they reported an age of first pneumonia at < 16 years or “As a child; age not known.” Subjects were classified as no childhood pneumonia if they reported no pneumonias, an age of first pneumonia ≥ 16 years, or if they did not indicated their first pneumonia was during childhood. Age sixteen was used to define pediatric pneumonia as this was when most subjects in the cohort started smoking (mean 16.9, standard deviation 4.6 years), which is concurrent with a rise in subjects reporting a first episode of pneumonia between ages 15-20.

Chronic bronchitis was defined by cough and phlegm production lasting more than three months per year for at least two years. COPD exacerbations were defined by use of antibiotics or systemic steroids. Severe COPD exacerbations required an emergency room visit or hospitalization. Cardiovascular disease (CVD) was defined by self-reported history of coronary artery disease, congestive heart failure, heart attack (MI), angioplasty, coronary artery bypass graft surgery, peripheral vascular disease, transient ischemic attack, or stroke [25]. Childhood asthma was defined as reported history of asthma diagnosed by a health professional with age of onset at < 16. COPD was defined based on post-bronchodilator forced expiratory volume in the first second (FEV_1_) to forced vital capacity (FVC) ratio < 0.7 with FEV_1_ < 80 % predicted, corresponding to Global Initiative for Chronic Obstructive Lung Disease (GOLD) Stages 2-4 [26]. Control smokers had normal spirometry, defined as FEV_1_/FVC ≥ 0.7 and FEV_1_ ≥ 80 %.

### Statistical analysis

Subjects with and without childhood pneumonia were compared by demographics, respiratory symptoms/diseases, lung function, and CT measurements. Statistical analysis was performed using R v3.1.1. Single variable analysis used chi-square tests, t-tests, or Wilcoxon rank sum tests. Multivariable regression analysis was performed, with most models adjusted for standard covariates of age, gender, race and smoking history. Additional covariates of FEV_1_ % predicted, height, body mass index, and CT scanner model were included for some analyses. Logistic regression reported odds ratios (OR) with 95 % confidence intervals (CI) and linear regression reported absolute differences (β) with standard errors (SE). Subjects with missing or unclassifiable responses were removed from specific analyses.

Regression analysis was repeated in three subsets. First, to assess the effect of asthma, the analysis was performed on subjects without childhood asthma. Second, the analysis was run on only subjects with childhood asthma. Finally, to assess the effect of recall bias, spirometry was analyzed in subjects who did not report a history of COPD or emphysema.

## Results

### Subject classification and characteristics

COPDGene includes 10,192 current and former smokers (Additional file 1: Figure S1). Thirty-six subjects were excluded, as it was not possible to classify their pneumonia history by questionnaire response. Of the 10,156 subjects included, 854 (8.4 %) reported childhood pneumonia. Of these, 405 subjects had COPD and 282 had normal spirometry. Of the 9,302 subjects without childhood pneumonia, 3,267 had COPD, and 4,097 had normal spirometry. Subjects with GOLD Stage 1 spirometry (FEV_1_/FVC < 0.7 with FEV_1_ ≥ 80 % predicted) or subjects with Preserved Ratio Impaired Spirometry (PRISm, FEV_1_/FVC ≥ 0.7 with FEV_1_ < 80 % predicted) were not included in COPD analysis; they were included in the other assessments [27].

Subjects with childhood pneumonia were older and more likely to be non-Hispanic white (Table 1). They were more likely to report living with a smoker in childhood, having a greater lifetime smoking intensity, were less likely to be current smokers, and had an increased number of lifetime pneumonia episodes. The distribution of age of first pneumonia can be seen in Additional file 1: Figure S2.

**Table 1:** Characteristics of Subjects With and Without History of Childhood Pneumonia

	Childhood Pneumonia		No Childhood Pneumonia		*p* Value^b^
	*N* = 854 (8.4 %)		*N* = 9302 (91.6 %)		
DEMOGRAPHIC					
Male gender (%)	437	(51.2 %)	4990	(53.6 %)	0.18
Mean age, years (SD)	61.7	(8.9)	59.4	(9.0)	<0.001^c^
Non-Hispanic white (%)	693	(81.1 %)	6073	(65.3 %)	<0.001
SMOKE EXPOSURE					
In-utero smoke exposure (%)^a^	206	(33.0 %)	2082	(30.2 %)	0.18
Lived with smoker in childhood (%)^a^	732	(85.7 %)	7618	(81.9 %)	0.006
Mean age started smoking, years (SD)	16.5	(4.4)	16.9	(4.7)	0.06
Pack-years of smoking (SD)	49.8	(28.4)	43.7	(24.6)	<0.001
Current smoking (%)	379	(44.4 %)	5011	(53.9 %)	<0.001
PNEUMONIA HISTORY					
Ever had pneumonia (%)	854	(100.0 %)	2979	(33.9 %)	<0.001
Diagnosed with pneumonia by healthcare provider (%)^a^	821	(96.1 %)	2920	(31.4 %)	<0.001
Pneumonia childhood age unknown (%)	378	(44.3 %)	0	(0.0 %)	<0.001
Age first pneumonia in years, mean (SD)^a^	7.7	(4.5)	42.5	(15.6)	<0.001
Lifetime pneumonia episodes (SD)^a^	3.9	(4.9)	2.5	(3.0)	<0.001

Abbreviations: *SD* standard deviation

^a^Subjects included are fewer than total subjects due to subject survey response being missing or unclassifiable

^b^Univariate analysis with chi-square or Wilcoxon rank sum test unless otherwise specified^c^ t test

### Respiratory symptoms and disease

Smokers with childhood pneumonia were more likely to develop COPD (Table 2). This remained robust when childhood asthma was added to the model. Childhood pneumonia was associated with increased chronic bronchitis, more frequent and severe COPD exacerbations in the year prior and increased frequency of co-morbid CVD (Table 3). These subjects were more likely to report asthma diagnosed by a healthcare provider and asthma onset in childhood. Childhood pneumonia was associated with worse disease-related quality of life with higher SGRQ, and more severe dyspnea with higher MMRC.

**Table 2:** COPD in Subjects With and Without History of Childhood Pneumonia

	Childhood Pneumonia		No Childhood Pneumonia		Impact of Childhood Pneumonia^a^		
	*N* = 687 (8.5 %)		*N* = 7364 (91.5 %)		OR	(95 % CI)	*p* Value^b^
COPD, GOLD 2-4	405	(59.0 %)	3267	(44.4 %)	1.40	(1.17, 1.66)	<0.001
COPD, GOLD 2-4 + adjusted for childhood asthma					1.30	(1.09, 1.55)	0.003

Abbreviations: *COPD* chronic obstructive pulmonary disease; *GOLD* Global Initiative for Chronic Obstructive Lung Disease

^a^Each row represents a separate regression model, odds ratio (OR) and 95 % confidence interval (CI) for logistic regression

^b^Covariates for all analyses = age at enrollment in years + gender + race + pack years

**Table 3:** Respiratory Symptoms and Disease in Subjects With and Without History of Childhood Pneumonia

	Childhood Pneumonia		No Childhood Pneumonia		Impact of Childhood Pneumonia^b^		
	*N* = 854 (8.4 %)		*N* = 9302 (91.6 %)		OR (95 % CI) or β (SE)^c^		*p* Value^d^
Chronic bronchitis (%)	214	(25.1 %)	1730	(18.6 %)	1.40	(1.18, 1.66)	<0.001^e^
Number of COPD exacerbations in past year (SD)	0.65	(1.2)	0.36	(0.9)	0.18	(0.03)	<0.001^f^
Had a severe COPD exacerbation in past year (%)	140	(16.4 %)	1063	(11.4 %)	1.28	(1.04, 1.58)	0.02^f^
Cardiovascular Disease (%)^a^	179	(21.0 %)	1455	(15.6 %)	1.20	(1.00-1.44)	0.047
Diagnosed with asthma by healthcare provider (%)^a^	239	(28.0 %)	1508	(16.3 %)	2.15	(1.83, 2.53)	<0.001
Childhood asthma (%)^a^	137	(16.0 %)	586	(6.3 %)	3.30	(2.68, 4.05)	<0.001
SGRQ Score, Total (SD)^a^	32.4	(24.0)	26.9	(22.8)	2.32	(0.67)	<0.001^g^
MMRC Dyspnea Scale, 0-4 (SD)^a^	1.6	(1.5)	1.3	(1.4)	0.12	(0.04)	0.006^g^

Abbreviations: *COPD* chronic obstructive pulmonary disease; *SD* standard deviation; *SGRQ* St. George’s Respiratory Questionnaire; *MMRC* Modified Medical Research Council

^a^Subjects included are fewer than total subjects due to subject survey response being missing or unclassifiable

^b^Each row represents a separate regression model

^c^Odds ratio (OR), 95 % confidence interval (CI) for logistic regression; beta coefficient (β), standard error (SE) for linear regression

^d^Covariates for all analyses = age at enrollment in years + gender + race + pack-years

^f^Additional covariates: ^e^current smoker; current smoker & FEV_1_ % predicted; ^g^FEV_1_ % predicted

**Fig. 1:**
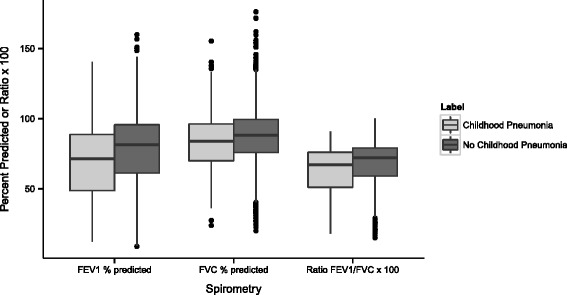
Lung Function. Decreased lung function with history of childhood pneumonia. Post-bronchodilator spirometry values are shown. Abbreviations: FEV1 = forced expiratory volume in the first second; FVC = forced vital capacity

**Fig. 2:**
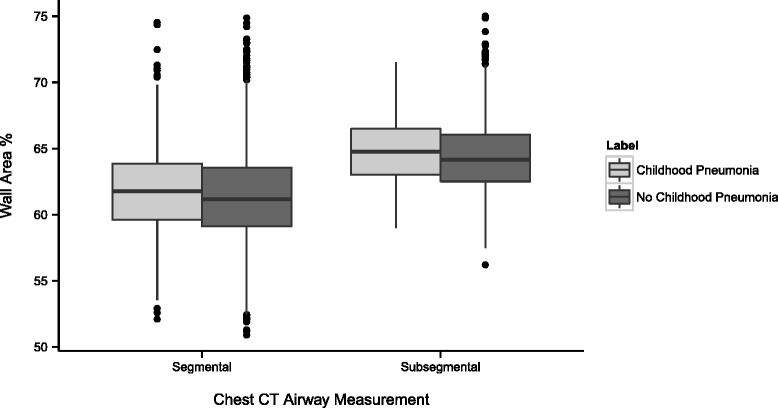
Chest CT. Airway changes on chest CT scans are associated with childhood pneumonia

Spirometry showed post-bronchodilator FEV_1_ % predicted, FVC % predicted and FEV_1_/FVC were all significantly lower in subjects with childhood pneumonia (Fig. 1, Table 4). In regression analysis, chest CT parameters related to airways disease were significantly increased in subjects with childhood pneumonia, with greater airway wall thickness in segmental and subsegmental airways and greater SRWA-Pi10 (Fig. 2, Table 5 and in the Additional file 1: Table S1). These subjects also had increased gas trapping. Multivariable analysis showed no difference in emphysema or total lung capacity measured by chest CT.

**Table 4:** Effect of Childhood Pneumonia on Lung Function

	Childhood Pneumonia	No Childhood Pneumonia	Impact of Childhood Pneumonia^a^		
	*N* = 850 (8.4 %)	*N* = 9245 (91.6 %)	β	SE	*p* Value^b^
FEV_1_ post-BD % predicted (SD)	69.1 % (25.7)	77.1 % (25.4)	−6.22	(0.88)	<0.001
FVC post-BD % predicted (SD)	82.7 % (18.6)	87.4 % (18.3)	−3.89	(0.65)	<0.001
FEV_1_/FVC post-BD (SD)	0.63 (0.17)	0.67 (0.16)	−0.02	(0.005)	<0.001^c^

Abbreviations: *FEV*_1_ forced expiratory volume in the first second; *FVC* forced vital capacity; *post-BD* post bronchodilator

^a^Each row represents a separate regression model, beta coefficient (β) and standard error (SE) for linear regression

^b^Covariate used for all analyses = pack years^c^Additional covariates = age at enrollment + gender + race + height

**Table 5:** Effect of Childhood Pneumonia on Chest CT Parameters

	Impact of Childhood Pneumonia^b^		
	β^c^	SE	*p* Value^d^
Wall Area %, Segmental	0.46	(0.12)	<0.001
Wall Area %, Subsegmental^a^	0.47	(0.16)	0.003
SRWA-Pi10	0.02	(0.005)	<0.001
Emphysema % (-950 HU)	0.18	(0.32)	0.57
Gas Trapping %, expiratory scan (-856HU)	1.97	(0.72)	0.006
Total Lung Capacity (L)	0.001	(0.04)	0.99

Abbreviations: *CT* computed tomography; *HU* Hounsfield units; *SRWA-Pi10* square root wall area of a hypothetical airway with 10mm internal perimeter

^a^Data only available for a limited portion of the cohort

^b^Each row represents a separate regression model

^c^Beta coefficient (β) and standard error (SE) for linear regression

^d^Covariates used = age at enrollment in years + gender + race + pack-years + body mass index + CT scanner model

### Sensitivity and recall analyses

To assess the effect of asthma, regression analysis was repeated in a subset of 9,405 subjects, excluding 723 with childhood asthma and 28 with unclassifiable childhood asthma status (Additional file 1: Table S2). Childhood pneumonia remained significantly associated with COPD (OR 1.24; 95 % CI 1.03-1.50). Other significant associations with childhood pneumonia were maintained, with the exception of the association with severe COPD exacerbations in the past year, which was attenuated.

A corresponding analysis was run including only 723 subjects with childhood asthma (Additional file 1: Table S3). In this subset, having childhood pneumonia showed a stronger association with COPD than in the cohort overall (OR 1.85; 95 % CI 1.10-3.18). The associations with post-bronchodilator percent predicted FEV_1_ and FVC remained significant. Other associations were no longer significant.

To assess recall bias, the regression analysis was repeated in a subset of 5,743 subjects who did not report COPD or emphysema diagnosis at enrollment (Additional file 1: Table S4). This included subjects with undiagnosed COPD and without COPD. Although prevalence of COPD was not significantly increased in this subset, both percent predicted FEV_1_ and FVC remained significantly lower in subjects with childhood pneumonia.

## Discussion

In adult smokers, a history of childhood pneumonia was associated with COPD and reduced lung function, with the greatest association in the subset of subjects with both pneumonia and asthma in childhood. Those with childhood pneumonia had increased chronic bronchitis, more frequent and severe COPD exacerbations, more CVD, increased dyspnea, and worse disease-related quality of life. There was a novel finding of greater airways disease present in chest CT scans of subjects with childhood pneumonia, supporting the idea that childhood disease is associated with long term structural differences in the lung and a distinct COPD phenotype. By comparison, there was no difference in emphysema.

The role of childhood pneumonia in COPD development has been investigated for over sixty years. Oswald surveyed 1000 adults with chronic bronchitis in London from 1951-53, finding 14.3 % reported childhood pneumonia compared to 6 % of controls [28]. In the 1970’s Burrows proposed that childhood respiratory infections are a risk factor for obstructive lung disease in adults, with an enhanced effect in smokers, based on decreased FEV_1_ and FEV_1_/FVC and increased chronic bronchitis in 415 subjects, mean age of 44.5, who reported childhood respiratory trouble at < 16 years [3]. Four subsequent investigations looked more closely at the relationship between childhood pneumonia in British subjects born between 1911-1935 and adult lung function at ages 34–74, each independently finding an association with decreased FEV_1_ and FVC, and suggesting an association between childhood pneumonia and COPD [4–7]. Only one of these five studies used post-bronchodilator data and none included COPD diagnosis in their outcomes [7].

The European Community Respiratory Health Survey assessed pre-bronchodilator lung function in subjects ages 20-44 in 1991-93 and then again 5-11 years later, showing risk of developing COPD was doubled in subjects with self-reported history of serious respiratory infection at < 5 years, and that this factor accounted for about 8 % of new cases [8, 9, 13]. Recent data from the Tucson Children’s Respiratory Study provided longitudinal post-bronchodilator lung function in 44 subjects born between 1980-84 with radiographically diagnosed pneumonia at ≤ 3 years, demonstrating an association with a persistent decrease in post-bronchodilator FEV_1_ and FEV_1_/FVC up to age 26 [10].

Compared to prior investigations, our study examines 10,192 United States smokers ages 45-80, including 854 subjects with childhood pneumonia. Our analysis supports the association between childhood pneumonia, reduced lung function in adulthood, and COPD. Our study is unique due to the older age of participants, the objective diagnosis of COPD by post-bronchodilator spirometry, and chest CT analysis. Additionally, few other studies have addressed this question in a population of this size and included assessment of the combined effect of pneumonia and childhood asthma.

Research into the association between childhood and adult respiratory disease is complicated by the inherent difficulty differentiating between diagnoses of childhood pneumonia, asthma, and other respiratory illnesses, which can overlap and evolve over time. Asthma is independently associated with both childhood pneumonia and adult COPD [10, 13, 29, 30]. More recent studies of long-term outcomes from childhood pneumonia have differentiated pneumonia from other respiratory illness and have accounted for asthma in their analyses, finding that the effect of childhood pneumonia on future lung function is greater than that of other childhood respiratory infections, and is robust to adjustments for childhood asthma [5–8, 10]. This is similar to our finding, where the association of childhood pneumonia with COPD persisted even after adjusting for or removing childhood asthmatics. Notably, we found that it was the combined effect of pneumonia and asthma in childhood had the greatest association with COPD.

Prior investigators have cited the possibility of impaired childhood lung growth and development playing a role in this association [2, 4–6]. Chest CT changes demonstrated in this analysis, with increased airways disease in subjects with childhood pneumonia, support this hypothesis. There are two potential explanations. The first is that childhood pneumonia may cause airways changes that increased risk for future disease. Alternatively, there may be an underlying developmental abnormality of the lung that increases risk for both childhood pneumonia and lung disease in adult smokers. While asthma has also been associated with airways disease on CT scans, the imaging associations in this analysis were maintained even in a subset analysis where childhood asthmatics were removed, suggesting that childhood pneumonia likely has an independent role [31]. It would be interesting to examine the extent of bronchiectasis in this population given the known associations between childhood pneumonia and bronchiectasis [32], however, standardized visual readings of the chest CT scans are not yet available in COPDGene.

### Limitations

The ideal study for examining the connection between childhood pneumonia and COPD would follow subjects from conception to death [33]. However, the challenges of such a study have forced researchers to take other approaches. Studies that use historical medical records paired with current cohorts available for lung function testing can be limited by selection bias [4–7]. Studies that follow childhood cohorts are limited by younger age at follow-up, especially given that the highest rates of COPD are in those over 65 [7, 10, 34]. An alternative method, employed by our study, was the collection of self-reported medical history from a cohort of adults, understanding that this does not include details such as gestational age, birth weight, childhood socio-economic status, and objective diagnostic tests for childhood pneumonia. This COPDGene Study assessment includes only smokers; therefore we could not address the effect of childhood pneumonia in non-smokers.

We acknowledge that our strategy may lead to potential recall bias, where adult subjects with respiratory disease may be more likely to recall childhood illness. This analysis included a separate assessment for recall bias, focusing only on subjects who did not report a known COPD diagnosis, and thus were less likely to be biased in recalling childhood respiratory problems. In this subset analysis, childhood pneumonia remained associated with reduced lung function. Therefore, it is unlikely that overall study results were influenced by recall bias. Childhood pneumonia was not associated with COPD in this analysis, though this is not surprising given that the subset includes nearly all those with normal lung function, while being most likely to remove subjects with more severe COPD.

Self-reported pneumonia is a potential source of misclassification, however, prior studies have shown that self-reported pneumonia diagnosis has relatively good agreement with the medical record [35]. Additionally, versions of the American Thoracic Society Questionnaire have previously been used to examine pneumonia history, and prior epidemiologic surveys examining the relationship have also used subject self-report [3, 9, 36, 37]. Random misclassification would be expected to bias towards null results, yet we still found a significant association.

## Conclusions

We found that the combination of pneumonia in childhood and smoking in adulthood is associated with COPD, increased respiratory symptoms, and reduced lung function. This was supported by novel findings of airways disease on chest CT scans. The greatest association with COPD was seen in those who had both pneumonia and asthma in childhood. Further research will be required to identify whether there are genetic associations that may play a role in determining a subtype of COPD that originates with childhood respiratory disease. In the meantime, medical providers have a valuable opportunity to reduce childhood pneumonias, especially among asthmatics, and to counsel patients about the increased risk from smoke exposure in those who have had pneumonia during childhood.

## Additional file

Additional file 1: Table S1. Chest CT Parameters for Subjects With and Without History of Childhood Pneumonia. **Table S2.** Effect of Childhood Pneumonia with Childhood Asthmatics Removed. **Table S3.** Effect of Childhood Pneumonia in Childhood Asthmatics Only. **Table S4.** Recall Assessment in Subjects Who Did Not Report Known COPD or Emphysema Diagnosis. **Figure S1.** Classification of subjects in cohort based on childhood pneumonia status. **Figure S2.** Distribution of age of first pneumonia in entire cohort (a) in subjects with a history of childhood pneumonia (b) and in subjects without a history of childhood pneumonia (c). Includes all subjects who reported an age of first pneumonia. (PDF 943 kb)

## Abbreviations

β: beta coefficient
CI: Confidence interval
COPD: Chronic obstructive pulmonary disease
CT: Computed tomography
CVD: Cardiovascular disease
FEV_1_: Forced expiratory volume in the first second
FVC: Forced vital capacity
GOLD: Global initiative for chronic obstructive lung disease
HU: Hounsfield units
MMRC: Modified Medical Research Council
OR: Odds ratio
post-BD: post bronchodilator
PRISm: Preserved ratio impaired spirometry
SE: Standard error
SGRQ: St. George’s Respiratory Questionnaire
SRWA-Pi10: Square root of wall area of a hypothetic airway of 10 mm internal perimeter
