# Supplementary material for: Childhood pneumonia increases risk for chronic obstructive pulmonary disease: the COPDGene study
Source: Respir Res. 2015 Sep 21;16(1):115. doi: 10.1186/s12931-015-0273-8 (PMC4578796; doi:10.1186/s12931-015-0273-8)
Supplement: Additional file 1: Table S1. — Chest CT Parameters for Subjects With and Without History of Childhood Pneumonia. Table S2. Effect of Childhood Pneumonia with Childhood Asthmatics Removed. Table S3. Effect of Childhood Pneumonia in Childhood Asthmatics Only. Table S4. Recall Assessment in Subjects Who Did Not Report Known COPD or Emphysema Diagnosis. Figure S1. Classification of subjects in cohort based on childhood pneumonia status. Figure S2. Distribution of age of first pneumonia in entire cohort (a) in subjects with a history of childhood pneumonia (b) and in subjects without a history of childhood pneumonia (c). Includes all subjects who reported an age of first pneumonia. (PDF 943 kb) [file 12931_2015_273_MOESM1_ESM.pdf]

Childhood pneumonia increases risk for chronic obstructive pulmonary disease: the COPDGene Study

Lystra P. Hayden, MD, MMSc, Brian D. Hobbs, MD, Robyn T. Cohen, MD, MPH, Robert A. Wise, MD, William Checkley, MD, PhD, James D. Crapo, MD, Craig P. Hersh, MD, MPH, on behalf of the COPDGene Investigators.

Supplemental Material

Tables

Table s1 Chest CT Parameters for Subjects With and Without History of Childhood Pneumonia

Chest CT Parameters	Childhood Pneumonia *N*		SD	No Childhood Pneumonia *N*		SD	p Value
Wall Area %, Segmental	*792*	61.9%	(3.3)	*8693*	61.4%	(3.3)	<0.001 [a]
Wall Area %, Subsegmental	*286*	64.8%	(2.6)	*2923*	64.3%	(2.6)	0.003 [a]
SRWA-Pi10	*768*	3.70	(0.14)	*8526*	3.68	(0.13)	0.01 [b]
Emphysema % (-950 HU)	*789*	7.2%	(9.7)	*8679*	6.1%	(9.6)	<0.001 [b]
Gas Trapping %, expiratory scan (-856HU)	*609*	27.0%	(21.4)	*6810*	22.1%	(20.3)	<0.001 [b]
Total Lung Capacity (L)	*789*	5.7	(1.4)	*8679*	5.5	(1.4)	0.004 [a]

Abbreviations: CT = computed tomography; HU = Hounsfield units. SRWA-Pi10 = square root wall area of a hypothetical airway with 10mm internal perimeter.
Univariate analysis with: [a] t-test; [b] Wilcoxon rank sum test.

**Table s2 Effect of Childhood Pneumonia with Childhood Asthmatics Removed**			
**Outcomes in N = 9,405 subjects, after removal of childhood asthmatics *** Childhood pneumonia N = 717 (7.6%); No childhood pneumonia N = 8688 (92.4%)	**Impact of Childhood Pneumonia [b]** **OR (95% CI) or β (SE) [c]**		**p Value**
**RESPIRATORY SYMPTOMS AND DISEASE**			
Chronic bronchitis	1.34	(1.12, 1.61)	0.002 [d,f]
COPD, GOLD 2-4	1.24	(1.03, 1.50)	0.02 [d]
Had a severe COPD exacerbation in past year	1.20	(0.94, 1.51)	0.14 [d,g]
Number of COPD exacerbations in past year	0.16	0.03	<0.001 [d,g]
SGRQ Score, Total [a]	2.00	0.71	0.005 [d,h]
MMRC Dyspnea Scale (0-4) [a]	0.14	0.05	0.003 [d,h]
**LUNG FUNCTION**			
FEV_1 post-BD (% predicted) [a]	-5.52	0.52	<0.001 [e]
FVC post-BD (% predicted) [a]	-3.31	0.70	<0.001 [e]
FEV_1/FVC post-BD (ratio x 100) [a]	-1.70	0.006	0.003 [d,h]
**CHEST CT PARAMETERS**			
Wall Area %, Segmental [a]	0.31	0.12	0.01 [d,j]
Wall Area %, Subsegmental [a]	0.41	0.17	0.01 [d,j]
SRWA-Pi10 [a]	0.01	0.005	0.04 [d,j]
Emphysema % (-950 HU) [a]	0.14	0.35	0.69 [d,j]
Gas Trapping %, expiratory scan (-856HU) [a]	1.79	0.78	0.02 [d,j]
Total Lung Capacity (L) [a]	0.008	0.04	0.84 [d,j]

Abbreviations: COPD = chronic obstructive pulmonary disease; GOLD = Global Initiative for Chronic Obstructive Lung Disease; SGRQ = St. George's Respiratory Questionnaire; MMRC = Modified Medical Research Council; FEV_1 = forced expiratory volume in the first second; FVC = forced vital capacity; post-BD = post bronchodilator; CT = computed tomography; HU = Hounsfield units. SRWA-Pi10 = square root wall area of a hypothetical airway with 10mm internal perimeter.

* From original 10,156 subjects, 723 were removed with childhood asthma and 28 were removed for unclassifiable asthma status.

[a] Subjects included are fewer than total subjects due to subject survey response being missing or unclassifiable. [b] Each row represents a separate regression model. Included subjects in each model are those with classifiable data for the parameter.

[c] Odds ratio (OR), 95% confidence interval (CI) for logistic regression; beta coefficient (β), standard error (SE) for linear regression.

[d] Covariates for analysis = age at enrollment in years + gender + race + pack-years. [e] Only covariate is pack-years.

Additional covariates: [f] current smoker; [g] current smoker + FEV_1 % predicted; [h] FEV_1 % predicted; [i] height; [j] body mass index + CT scanner model.

Table s3 Effect of Childhood Pneumonia in Childhood Asthmatics Only			
**Outcomes in N = 723 subjects, only those with childhood asthma**<br>Childhood pneumonia N = 137 (18.9%); No childhood pneumonia N = 586 (81.1%)	**Impact of Childhood Pneumonia [b]**<br>**OR (95% CI) or β (SE) [c]**		**p Value**
**RESPIRATORY SYMPTOMS AND DISEASE**			
Chronic bronchitis	1.14	(0.13, 3.56)	0.56 [d,f]
COPD, GOLD 2-4	1.85	(1.10, 3.18)	0.02 [d]
Number of COPD exacerbations in past year	0.05	0.12	0.70 [d,g]
Had a severe COPD exacerbation in past year	1.22	(0.76, 1.94)	0.41 [d,g]
SGRQ Score, Total [a]	-0.44	2.02	0.83 [d,h]
MMRC Dyspnea Scale (0-4) [a]	-0.12	0.13	0.33 [d,h]
**LUNG FUNCTION**			
FEV_1 post-BD (% predicted) [a]	-6.24	2.35	0.008 [e]
FVC post-BD (% predicted) [a]	-4.66	1.79	0.009 [e]
FEV_1/FVC post-BD (ratio x 100) [a]	-0.01	0.01	0.64 [d,i]
**CHEST CT PARAMETERS**			
Wall Area %, Segmental [a]	0.39	0.33	0.25 [d,j]
Wall Area %, Subsegmental [a]	0.32	0.48	0.51 [d,j]
SRWA-Pi10 [a]	0.02	0.02	0.27 [d,j]
Emphysema % (-950 HU) [a]	0.42	0.77	0.58 [d,j]
Gas Trapping %, expiratory scan (-856HU) [a]	0.76	1.93	0.70 [d,j]
Total Lung Capacity (L) [a]	-0.08	0.01	0.43 [d,j]

Abbreviations: COPD = chronic obstructive pulmonary disease; GOLD = Global Initiative for Chronic Obstructive Lung Disease; SGRQ = St. George's Respiratory Questionnaire; MMRC = Modified Medical Research Council; FEV_1 = forced expiratory volume in the first second; FVC = forced vital capacity; post-BD = post bronchodilator; CT = computed tomography; HU = Hounsfield units. SRWA-Pi10 = square root wall area of a hypothetical airway with 10mm internal perimeter.

[a] Subjects included are fewer than total subjects due to subject survey response being missing or unclassifiable.

[b] Each row represents a separate regression model. Included subjects in each model are those with classifiable data for the parameter.

[c] Odds ratio (OR), 95% confidence interval (CI) for logistic regression; beta coefficient (β), standard error (SE) for linear regression.

[d] Covariates for analysis = age at enrollment in years + gender + race + pack-years. [e] Only covariate is pack-years.

Additional covariates: [f] current smoker; [g] current smoker + FEV_1 % predicted; [h] FEV_1 % predicted; [i] height; [j] body mass index + CT scanner model.

**Table s4 Recall Assessment in Subjects Who Did Not Report Known COPD or Emphysema Diagnosis**					
	**Childhood Pneumonia** N = 374 (6.5%)	**No Childhood Pneumonia** N = 5369 (93.5%)	**Impact of Childhood Pneumonia** [b] **OR (95% CI) or β (SE)**		**p Value** [c]
COPD, GOLD 2-4 (%) [a]	54 (14.4%)	698 (13.0%)	1.03	(0.74, 1.41)	0.85 [d]
FEV_1 post-BD % predicted [a]	87%	89%	-2.29	(0.91)	0.01
FVC post-BD % predicted [a]	91%	93%	-1.80	(0.82)	0.03
FEV_1/FVC post-BD (ratio x 100) [a]	0.74	0.75	-0.004	(0.005)	0.35 [d,e]

Abbreviations: COPD = chronic obstructive pulmonary disease; GOLD = Global Initiative for Chronic Obstructive Lung Disease; FEV_1 = forced expiratory volume in the first second; FVC = forced vital capacity; post-BD = post bronchodilator.
[a] Subjects included are fewer than total subjects due to subject survey response being missing or unclassifiable.
[b] Each row represents a separate regression model. Beta coefficient (β) and standard error (SE) for linear regression.
[c] Covariate used for all analyses is pack years. [d] Additional covariates: gender + age at enrollment + race; [e] + height.

Figures

Figure s1 Subject Classification

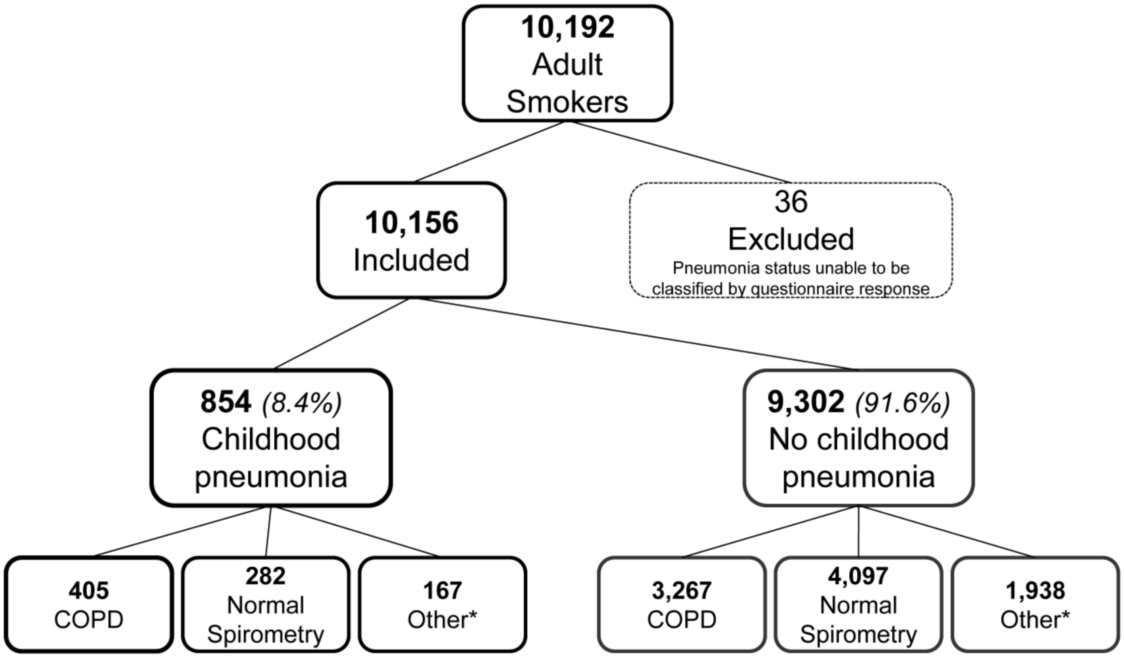

Figure s1 Classification of subjects in cohort based on childhood pneumonia status.

*Includes subjects with GOLD Stage 1 ($FEV_1/FVC < 0.7$ with $FEV_1 \geq 80\%$ predicted) or Preserved Ratio Impaired Spirometry (PRISm, $FEV_1/FVC \geq 0.7$ with $FEV_1 < 80\%$ predicted

Figure s2 Pneumonia Age Distribution

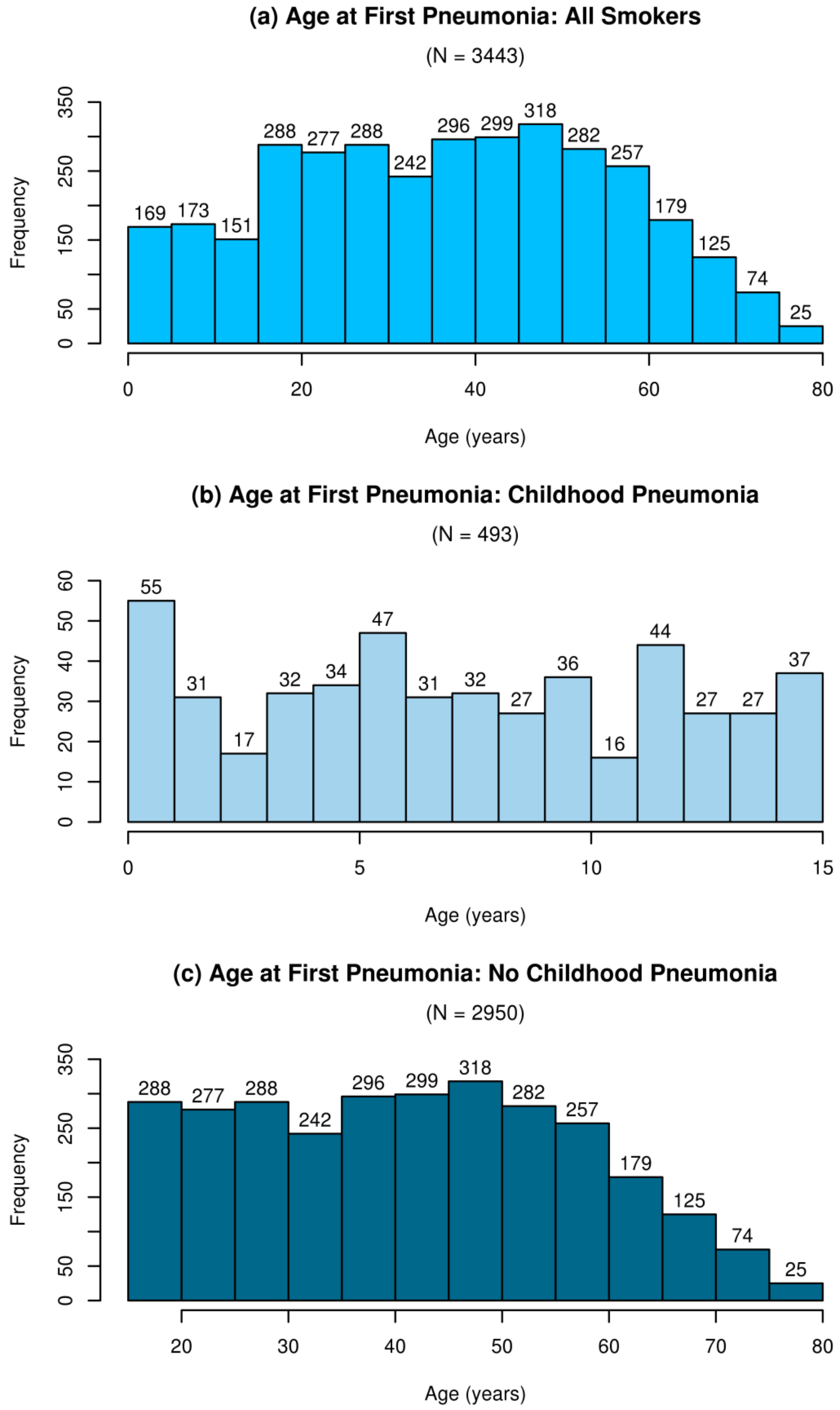

Figure s2 Distribution of age of first pneumonia in entire cohort (a) in subjects with a history of childhood pneumonia (b) and in subjects without a history of childhood pneumonia (c). Includes all subjects who reported an age of first pneumonia.
